# Unsteady walking as a symptom in type 2 diabetes mellitus: independent association with depression and sedentary lifestyle and no association with diabetic neuropathy

**DOI:** 10.1590/1414-431X20186605

**Published:** 2018-03-26

**Authors:** L.S. Dias, O.H. Nienov, C.F. Goelzer, H. Schmid

**Affiliations:** 1 Programa de Pós-Graduação em Ciências da Saúde, Ginecologia e Obstetrícia, Faculdade de Medicina, Universidade Federal do Rio Grande do Sul, Porto Alegre, RS, Brasil; 2 Programa de Pós-Graduação em Ciências da Saúde, Universidade Federal de Ciências da Saúde de Porto Alegre, Porto Alegre, RS, Brasil; 3 Serviço de Endocrinologia, Hospital Santa Casa de Misericórdia de Porto Alegre, Porto Alegre, RS, Brasil; 4 Departamento de Medicina Interna, Hospital de Clínicas de Porto Alegre, Porto Alegre, RS, Brasil

**Keywords:** Diabetic neuropathy, Depression, Unsteady walking, Lifestyle

## Abstract

The purpose of this study was to look at the determinants of the unsteady walking (UW) symptom in patients with type 2 diabetes mellitus (T2DM) by defining if UW and/or the Diabetic Neuropathy Symptoms Score (DNSS) are associated with positive scores in Beck's Depression Inventory (BDI) and with a positive Michigan Neuropathy Screening Instrument score (MNSI). We evaluated 203 T2DM patients without visible gait disturbances. They were divided into UW (+) and UW (−) or DNSS (+) and DNSS (−) according to symptoms. We found a prevalence of 48.3% for UW (+) and of 63% for DNSS (+) in our sample. In univariate analysis, the presence of UW was significantly associated with waist circumference (P=0.024), number of comorbidities (P=0.012), not practicing physical exercise (P=0.011), positive BDI score (P=0.003), presence of neuropathic symptoms by the MNSI questionnaire (P<0.001), and positive diabetic neuropathy screening by MNSI (P=0.021). In multivariate analysis, UW (used as a dependent variable) was independently associated with a positive BDI score (P<0.001; 95%CI=1.01-1.03), T2DM duration (P=0.023; 95%CI=1.00–1.03), number of co-morbidities (P=0.032; 95%CI=1.01–1.37), and a sedentary lifestyle (P=0.025; 95%CI=1.06–2.5). The UW symptom and a positive DNSS are more closely related to a positive score for depression than to presence of neuropathy in T2DM.

## Introduction

Since a direct relationship between neuropathic and depressive symptoms has been demonstrated in different studies (1–3) the use of scales for screening neuropathy and depression in diabetic patients should be cautiously interpreted. Vileikyte et al. suggest that the symptom of unsteadiness in walking (UW) has a biological basis that could provide a possible explanation for this finding, since it is common to both depression in type 2 diabetes mellitus (T2DM) and peripheral polyneuropathy (PPN) (3). By collecting objective data, some authors have also shown that walking impairment occurs as a late complication of diabetes, primarily in people with established neuropathy (4,5).

The most frequently used instruments for screening diabetic neuropathy are the Michigan Neuropathy Screening Instrument (MNSI) (6) and the Neuropathy Disability Score (NDS) plus the Neuropathy Symptom Score (NSS) (7). All of them provide a good identification of people with large fiber disease. The instruments do not have questions or measurements related to UW (8).

In 2002, Meijer et al. (9) validated a questionnaire that had an item about UW. It is fast and easy to perform because it evaluates only answers to four questions (9,10). The authors found a high predictive value when screening for PPN. It was called the Diabetic Neuropathy Symptoms Score (DNSS) and according to the authors, it has an 88% correlation with the NSS (10). Since the instrument contains a question specifically related to UW, we thought that a positive answer to the item might be associated with depression and PPN. The purpose of this study was to look at the determinants of UW in patients with T2DM by investigating if UW is associated with neuropathy or depression.

## Material and Methods

A cross-sectional and consecutive study in patients with T2DM was conducted at the Centro de Educação e Pesquisa em Diabetes e Obesidade, Santa Casa de Misericórdia de Porto Alegre, RS, Brasil. All patients were undergoing exams and consultations for the treatment of diabetes and its complications at the Diabetes Mellitus/Endocrinology outpatient clinic belonging to the Sistema Único de Saúde (SUS, the Brazilian National Health Service) of this hospital. The patients were evaluated during their routine visits by two examiners who had no knowledge of their co-morbidities. We used the following inclusion criteria: patients with DM according to the fasting plasma glucose of the American Diabetes Association criteria (11) for more than 1 year, patients older than 18 years regardless of gender, patients who had the test results of consultations done in the previous month, and patients who wished to participate. The exclusion criteria were patients with any kind of lower limb amputation and plantar ulcer, evident gait disturbances on physical exam, orthostatic hypotension, legal blindness, dizziness, a positive Romberg test, hypothyroidism (TSH >6 mU/L), B12 vitamin deficiency (vitamin B12 <210 pg/mL), history of alcohol abuse (according to the CAGE questionnaire) (12), history of use of sedatives, tranquilizers, antiepileptic, and phenothiazine drugs, neuropathy related to causes other than DM, high degree (3 or 4) of renal failure, acute liver disease, symptomatic cardiovascular disease, asthma, pain related to arthritis, migraine headaches, lactating and pregnant women, patients who were unable to answer the written questions or had a history of inner ear problems, Parkinson's disease, Alzheimer disease, and previous cancer (except basal cell skin cancer).

This study was approved by the Ethics Committee of Santa Casa de Porto Alegre (No. 147/2012) and by the Ethics Committee of Universidade Federal de Ciências da Saúde de Porto Alegre (No. 1452/11). All patients signed a written informed consent to participate.

On the day of the evaluation, data were obtained about age, life style habits, body weight and height, body mass index (BMI), and waist circumference. All participants answered the CAGE questionnaire to define the presence of alcoholism and patients were submitted to a brief neurological assessment that included strength and tone of lower limbs, coordination, standing, and gait. They were screened for simple postural control and for proprioceptive and vestibular system dysfunction when the eyes were closed. Patients were considered sedentary if they did not practice at least 150 min/week of moderate to high intensity exercise (13).

Forty-five of the 248 patients invited to participate were excluded: 13 because their data were incomplete; 8 because their TSH levels were higher than 6 mU/L; 5 because they had vitamin B12 deficiency; 7 because they had a CAGE positive questionnaire, and 12 because they declined to participate.

Laboratory data (obtained in the last month of neuropathy evaluation) was collected from the patients' hospital records. Metabolic syndrome was defined according to the International Diabetes Federation criteria (14).

Depressive symptoms were assessed by the Brazilian version of the Beck Depression Inventory (BDI) (15) translated and validated into Portuguese (16,17). Depressive symptoms were classified according to the number of points as minimal (0–9), mild (10–18), moderate (19–29), or severe (30–63) (15).

The instrument proposed for defining the presence of neuropathy was the MNSI (6). A physician from our group was trained to perform this test at the University of Michigan and this physician trained two of our biomedical professionals. The two professionals performed all the tests and if there was a disagreement, the physician examined the patient and gave the final decision.

The questionnaire that was used to assess the correlation with depression and the presence of neuropathy was the DNSS, which consists of answering yes or no to the following items: 1) unsteadiness on walking (*desequilíbrio ao caminhar* in Portuguese), 2) pain, burning, or aching legs or feet, 3) prickling sensations in the legs or feet, and 4) numbness of the legs or feet. Presence of one symptom was scored as 1 point, absence as 0, and the maximum score was 4 points. The test was considered positive when at least one point was recorded (9,10). The questionnaire was not validated in Portuguese but only the first question (UW) completely differs from the questions of the NDS questionnaire, which is validated.

### Theory and calculation

In order to define the sample size, we conducted a pilot study with 43 diabetic subjects. The results showed that 21.18% of the patients had polyneuropathy, 62.8% had depression symptoms. We used the Program for Epidemiologists (PEPI) version 4.0 to calculate the size of the population sample. For a significance level of 5%, 90% power, an estimated prevalence of 72% of a positive DNSS, and a 25% difference between groups we found 174 to be the appropriate number of patients. By adding 10% for multivariate analysis, the minimum number of patients was 192.

The patients were divided into two groups, according to their answers to the DNSS questionnaire: DNSS (+) and DNSS (−); or to the first question as UW (+) and UW (−). Quantitative data are reported as the mean or median and qualitative data as frequency. Categorical data were tested using the chi-square test or Fisher's exact test when appropriate. Continuous variables were tested using the Student's *t*-test when there was a normal distribution of data or the Mann-Whitney test when the distribution was not normal. When a P≤0.05 was found, it was accepted that there was a significant difference.

Finally, using the software SPSS for Windows version 18 (USA), we performed a multiple Poisson regression analysis to evaluate which of the factors were independently associated with the occurrence of positive answers to UW and to the DNSS. The association of measures was evaluated by odds ratio and 95%CI. Variables that showed a P value of less than 0.2 in the univariate model were included in the model.

## Results

Among the T2DM patients evaluated, the UW prevalence was 48.3% and the DNSS (+) prevalence was 63%. All patients classified as having neuropathy by the physical exam (22% of the total sample), had at least two symptoms of diabetic neuropathy according to their responses to the questionnaire (data not shown). The co-morbidities presented by the patients were partial loss of vision in 35% (71 patients), cardiovascular disease in 28% (57 patients), foot ulcers (not plantar, not painful) in 2.5% (5 patients), 15% (30 patients) had more than one of these co-morbidities.

Table 1 shows the characteristics of the patients according to the groups. Mean age, level of education, BMI, waist circumference, blood pressure, heart rate, HbA1C, glucose, plasma lipids, creatinine, and number of medications used did not differ between the two groups.

**Table 1.** Demographic, anthropometric, and clinical characteristics of 203 patients with type 2 diabetes mellitus assessed for the presence of the symptom of unsteady walking (UW).

	UW (+) group (n=98)	UW (−) group (n=105)	P value
Age (years)	61.9±8.6	60.5±9.4	0.283^a^
BMI (kg/m^2^)	30.3±5.1	29.1±6.0	0.126^a^
Waist circumference (cm)	103.6±10.7	99.3±15.5	0.024^a^*
Level of education (years)	5 (3–8)	5 (3.5–8)	0.994^b^
Duration of DM (years)	12 (7.8–20)	10 (6–15)	0.067^b^
SBP (mmHg)	146.2±26.9	141.1±22.2	0.143^a^
DBP (mmHg)	82.5±19.1	82.6±11.1	0.979^a^
HR (bpm)	72.9±11.1	74.3±12.1	0.425^a^
HbA1c (%)	7.9±1.9	8.1±2.2	0.522^a^
Fasting glycemia (mg/dL)	159.4±64.8	162.5±74.2	0.751^a^
Total-cholesterol (mg/dL)	178.0±51.5	179.8±39.9	0.794^a^
HDL-cholesterol (mg/dL)	48.7±11.8	50.2±14.4	0.411^a^
LDL-cholesterol (mg/dL)	95.7±41.4	95.2±29.3	0.914^a^
Triglycerides (mg/dL)	162 (109–222)	142.5 (93–186)	0.091^b^
Creatinine (mg/dL)	1.1 (0.9–1.25)	1.1 (0.9–1.2)	0.855^b^
Comorbidities (n)	1 (0–1)	0 (0–1)	0.012^b^*
Medications (n)	6.2±2.1	5.7±2.0	0.059^a^

Data are reported as means±SD or median and 25 and 75% quartiles in parentheses. BMI: body mass index; SBP: systolic blood pressure; DBP: diastolic blood pressure; HR: heart rate; HbA1c: blood glycosylated hemoglobin levels; HDL: high-density lipoprotein; LDL: low-density lipoprotein. *P<0.05: ^a^Student's *t*-test; ^b^Mann-Whitney test.

Both groups also did not differ according to the percentage of female gender, number of blacks *vs* whites, percentage of patients with obesity, number of patients with increased plasma LDL-cholesterol and triglycerides, decreased HDL-cholesterol levels and increased waist circumference, number of patients with metabolic syndrome, number of smoking patients, and number of patients who had a sedentary life style (Table 2).

**Table 2.** Univariate analysis of demographic, anthropometric, and clinical characteristics of 203 patients with type 2 diabetes mellitus assessed for the presence of the symptom of unsteady walking (UW).

	UW (+) group (n=98)	UW (−) group (n=105)	P value
Female gender (n, %)	63 (64.3)	69 (65.7)	0.947
Caucasian (n, %)	78 (79.6)	76 (72.4)	0.300
Obesity (n, %)	51 (52.0)	44 (41.9)	0.192
MS (n, %)	89 (90.8)	92 (88.5)	0.751
Dyslipidemia (%)	83 (84.7)	81 (77.1)	0.235
Increased blood pressure (n, %)	92 (93.9)	90 (85.7)	0.093
High Total-cholesterol (n, %)	19 (20.2)	29 (28.4)	0.242
Low HDL-cholesterol (n, %)	56 (59.6)	54 (53.5)	0.475
High LDL-cholesterol (n, %)	36 (38.3)	44 (43.6)	0.548
Hypertriglyceridemia (n, %)	54 (57.4)	44 (44.0)	0.084
High HbA1c (n, %)	77 (79.4)	83 (81.4)	0.861
High fasting glycemia (n, %)	84 (85.7)	92 (87.6)	0.847
High waist circumference (n, %)	91 (92.9)	92 (88.5)	0.751
Smoking (n, %)	11 (11.2)	6 (5.7)	0.245
Sedentary lifestyle (n, %)	83 (84.7)	72 (68.6)	0.011*

Data are reported as number and percentage of patients with the condition described in each group. MS: metabolic syndrome; HDL-cholesterol: high-density lipoprotein cholesterol; LDL-cholesterol: low-density lipoprotein cholesterol; HbA1c: blood glycosylated hemoglobin levels. *P<0.05 (chi-square test).

Table 3 shows the presence of neuropathy (MNSI), values of the BDI score of the groups, and the number of patients according to the four degrees of depression. Table 3 also shows the univariate analysis for depression and for PPN screening. The median of the BDI score (10 *vs* 7; P=0.003) was increased in the UW (+) group of patients. Median values of the MNSI score (physical exam) were also increased for the UW (+) group (2 *vs* 1; P<0.021).

**Table 3.** Scores for depression and neuropathic symptoms of 203 patients with type 2 diabetes mellitus assessed for the presence of the symptom of unsteady walking (UW).

	UW (+) group (n=98)	UW (−) group (n=105)	P value
BDI Score	10 (6–20)	7 (3–14)	0.003*^a^
Neuropathic physical exam (MNSI)	2 (1–3)	1 (0–2)	0.021*^a^
Neuropathy prevalence (MNSI) (n, %)	26 (26.5)	18 (17.1)	0.147^b^
Previous depression diagnosis (n, %)	2 (2.0)	9 (8.6)	0.081^c^
Depressive symptoms (BDI) (n, %)			
Minimum depressive symptoms (0–9)	46 (46.9)	63 (60.0)	0.085^b^
Mild depressive symptoms (10–18)	25 (25.5)	26 (24.8)	1.000^b^
Moderate depressive symptoms (19–29)	20 (20.4)	14 (13.3)	0.246^b^
Severe depressive symptoms (30–63)	7 (7.1)	2 (1.9)	0.092^c^

Data are reported as the medians and interquartile range or number and percentage of patients with the condition described in each group. BDI: Beck Depression Inventory; MNSI: Michigan Neuropathy Screening Instrument. *P<0.05 (^a^Mann-Whitney test; ^b^chi-square test; ^c^Fisher's exact test).

Some of the variables were significant (P*<*0.05) or presented a trend for significance (P*<*0.2) in univariate analysis. Therefore, to identify the extent of the contribution of the variables that could be associated with the presence of UW, a new table was built with two models of multiple regression analysis (Table 4). The results showed a significant positive association of the presence of UW with DM duration (95%CI=1.00–1.03; P*=*0.023), number of co-morbidities (95%CI=1.02–1.37; P=0.025 and 95%CI=1.01–1.37; P=0.032), sedentary lifestyle (95%CI=1.11–2.59; P*=*0.014 and 95%CI=1.63 (1.06–2.50), previous depression diagnosis (95%CI=0.09–0.98; P=0.047), and BDI score (95%CI=1.01–1.03; P*<*0.001 and 95%CI=1.01–1.03; P<0.001). The physical exam of the MNSI was not associated with having the UW symptom.

**Table 4.** Multiple Poisson regression analysis performed to evaluate factors independently associated with the symptom of unsteady walking (UW).

Variables	Model 1		Model 2	
	PR (95%CI)	P value	PR (95%CI)	P value
DM Duration (years)	1.01 (1.00–1.03)	0.050*	1.02 (1.00–1.03)	0.023*
BMI (kg/m^2^)	1.01 (0.99–1.04)	0.366	NA	NA
Waist circumference (cm)	NA	NA	1.01 (0.99–1.02)	0.064
High blood pressure	1.44 (0.71–2.92)	0.320	1.43 (0.70–2.91)	0.329
Hypertriglyceridemia	1.16 (0.87–1.54)	0.312	1.17 (0.87–1.56)	0.292
Number of Comorbidities	1.18 (1.02–1.37)	0.025*	1.18 (1.01–1.37)	0.032*
Sedentary lifestyle	1.70 (1.11–2.59)	0.014*	1.63 (1.06–2.50)	0.025*
Previous depression	0.29 (0.09–0.98)	0.047*	0.31 (0.09–1.04)	0.059
BDI score	1.02 (1.01–1.03)	<0.001*	1.02 (1.01–1.03)	<0.001*
Physical exam (MNSI)	1.04 (0.97–1.12)	0.250	1.04 (0.97–1.12)	0.271

PR: prevalence ratio; DM: diabetes mellitus; BMI: body mass index; NA: not applicable; BDI: Beck Depression Inventory; MNSI: Michigan Neuropathy Screening Instrument. *P<0.05.

When the last three questions of the DNSS were used as the dependent variable, levels of triglycerides, smoking occurrence, and BDI score were correlated to it, but not to the Michigan Physical Score (data not shown).

## Discussion

DNSS is a simple questionnaire that was created as a method for measuring symptoms of neuropathy (9,10). Since it has questions that evaluate UW, a problem that has been related to both depression and PPN, we decided to evaluate whether the DNSS and the UW symptom are more closely related to PPN or depression.

In 2009, Vileikyte et al. (18) presented data about predictors of depressive symptoms in T2DM with PPN and showed that unsteadiness, which can be generated by the presence of polyneuropathy, is the symptom that is most strongly associated with depression in individuals with PPN. Since UW can also occur as a result of other problems, the conclusion of Ostrowska et al. (19) was that if this question is included in a questionnaire for evaluating PPN it would be important to have other specific questions to differentiate it from other causes.

In the present study, we evaluated the UW symptom and assessed its correlation to the presence of symptoms of depression and PPN. In a univariate analysis, our data showed that diabetes duration, as well as a measure of PPN (the MNSI), and a measure of depression (the BDI Score) were associated with a positive UW. However, when a multiple Poisson regression analysis was performed to evaluate which factors were independently associated with UW using two models, duration of diabetes, number of co-morbidities, sedentary lifestyle, and the Beck score were all independently associated, and there was no association with the physical exam of the MNSI. The strongest association was observed with a sedentary lifestyle, which means that patients who confirmed a feeling of UW were less physically active than the group without the UW symptom. It was impossible to define if this occurred because these people had gait problems not detected in our exam and consequently could not exercise or if they were unsteady on walking because they were not physically active and consequently had some degree of postural hypotension.

Since we assessed gait only by inspection and did not evaluate patients with more objective tests related to gait, it is impossible to tell if they had abnormal tests of motor performance during walking or not. In some studies, patients with depression symptoms, as well as patients with Parkinson's disease, have slowed motor performance and other motor disabilities (20,21). However, in those studies, the presence of PPN and UW were not explored. In a study in patients with Parkinson's disease and patients with depression, imaging evaluations revealed shrunken basal ganglia and changes in dopamine-dependent neural circuits (21,22). Thus, we think that these abnormalities could also be present in patients with diabetes who have depression.

Gait abnormalities (and maybe UW) can be categorized according to the level of the deficit in the nervous system, i.e., low, middle, and high. Low deficits include peripheral sensory and motor defects, middle deficits include spasticity, parkinsonism, and ataxia, and high deficits include slowed cognition and fear of falling (23). In the present study, we tried to exclude patients with middle deficits because they were not related to DM. The results showed that the symptom of UW was related more to a high deficit (depression symptoms) than to a low deficit (peripheral sensory polyneuropathy) in patients with DM.

In a study published by Ostrowska et al. (19), people over the age of 65 years were evaluated to find risk factors for falls. They found that the group with the lowest number of points in an ability test (Berg balance test) had all the risk factors for falls: postural instability, previous falls, and no physical activity. Considering these facts, we might speculate that patients who had UW were also more prone to the symptom because they had a more sedentary life.

The American Geriatrics Society and British Geriatrics Society recommend that all adults older than 65 years should be screened annually for a history of falls or balance impairment (24). An individualized risk assessment should be performed, with a corresponding multifactorial intervention for those who report a single fall and have UW, two or more falls, difficulties with gait or balance, or seek medical attention because of a fall. The following components should be included in multifactorial interventions: exercise, balance, strength, and gait training. With these recommendations, it is expected that the UW symptom could decrease, but some studies found a greater risk of falling with exercise (25).

Since the literature has many questionnaires that have been validated for the diagnosis of depression but not of neuropathy, we think that our study is useful in indicating that the questionnaire proposed by Meijer et al. (9) to diagnose PPN might not yield reliable results because it is more closely related to the depression that frequently occurs in these patients. Furthermore, we think that there is a potential use for this questionnaire in the follow-up of T2DM treated for depression and UW since unsteadiness on walking is a disability that should receive more attention in the education of both healthcare providers and patients (24).

We should also consider that UW could result from a PPN that was just beginning, from an incipient PPN that cannot yet be detected by physical exams usually used for this purpose or from another undetected incipient systemic disease. If this is the case, our patients could have secondary depression because they suffer from UW or other symptoms. Very recent studies agree that walking impairment might begin before the onset of large fiber neuropathy in diabetes (26,27) and suggest that small, rather than large fiber neuropathy may be a contributing factor.

The use of new instruments for detecting small fiber PPN, with simultaneous evaluation of depression scale scores and questions about UW in new studies, should answer whether the UW symptom is related not only to depression, but also to small fiber neuropathy.

In the present study, presence of UW and a positive DNSS are more closely related to a positive score for depression than to the presence of neuropathy in T2DM. The UW symptom was found to be independently associated with a condition that cannot be changed, which is the number of co-morbidities that diabetic patients present as well as with a condition that can be changed, the sedentary lifestyle.
